# Running into trouble with soy: A case report and review of our shopping carts

**DOI:** 10.1016/j.jacig.2024.100321

**Published:** 2024-08-05

**Authors:** Fionnuala Cox, Khairin Khalib, Mary Keogan

**Affiliations:** Department of Immunology, Beaumont Hospital, Dublin, Ireland

**Keywords:** Food allergy, soy allergy, food-dependent exercise-associated anaphylaxis, storage protein allergy, component-resolved diagnostics

## Abstract

Soy-dependent exercise-induced anaphylaxis is likely underdiagnosed and potentially on the rise. As soy gains popularity in Western diets, we highlight it as a hidden allergen in a variety of processed foods, including those marketed toward exercise enthusiasts.

Food-dependent exercise-induced anaphylaxis can present a diagnostic challenge, especially when the triggering allergen is consumed unknowingly. Although well-known triggers such as wheat, shellfish, and nuts are well documented, soy-dependent exercise-induced anaphylaxis (SDEIA) remains less commonly described.^1^ We present a unique case of SDEIA and provide a useful guide to potential hidden triggers.

A 16-year-old girl developed anaphylaxis following exertion 1 hour after a meal of breaded chicken and French fries. Her symptoms of throat irritation, rhinorrhoea, periorbital edema, and airway compromise were promptly treated with inhaled salbutamol, intramuscular adrenaline (300 μg × 2), and an antihistamine.

Three months after the event, the patient was avoiding peanut because of a known childhood allergy, would regularly eat a variety of foods peri-exercise, and had since tolerated a similar breaded chicken meal without having exercised. Over the subsequent 12 months, she experienced anaphylaxis after exertion in response to foods normally tolerated at rest (ie, a protein bar, granola, and a smoothie). Her specific IgE level, as measured by ImmunoCAP (Thermo Fisher Scientific, Waltham, Mass), confirmed peanut sensitization (37.5 kU/L [an *Arapis hypogaea* level of 24 kU/L]). Her levels of sensitization to specific IgE to soybean components (1.06 kU/L) were measured by Immuno-solid-phase allergen chip (Thermo Fisher Scientific) and demonstrated sensitization to the soy storage protein *Glycine max* [Gly m] 6 (3 Immuno-solid-phase allergen chip standardized units). The results of skin testing with a commercial soy extract and soy milk were negative. The soy extract used for testing was manufactured by Inmunotek (Madrid, Spain).

A review of the culprit foods revealed the presence of hydrolyzed soy protein (a bulking component in the breaded chicken) and granola; in addition, isolated soy protein was a primary ingredient in the protein bar. The patient’s clinical history and investigations were robust enough to confirm SDEIA. A food-exercise challenge was not deemed necessary. Complete soy avoidance was a shared decision owing to severity of the patient’s reactions and her regular involvement with sports. Adrenaline autoinjectors were prescribed, and management of the patient’s allergy focused on food label literacy, highlighting processed foods that can contain “hidden” soy and navigating eating outside the home. The patient was advised not to exercise alone or in isolated areas.

This case demonstrates soy as a hidden allergen and cause of food-dependent exercise-induced anaphylaxis. Gly m 6 sensitization has been described in a number of SDEIA cases, primarily in Asia,^2^ whereas reports in Western countries are sparse. Patients often experience several reactions to seemingly unrelated foods before a diagnosis is confirmed.^3^ Similar to what occurred in our patient, SDEIA is mostly recognized in those with peanut allergy who are also sensitized to the storage proteins Gly m 5 or Gly m 6, and the triggers include tofu or soy protein hidden in processed foods (Table I).^3^, ^4^, ^5^ Commercial skin testing is often unhelpful.^3^ Separately, SDEIA can occur in the context of birch pollen allergy in patients sensitized to the more labile soy pathogenesis-related protein 10 (PR-10) Gly m 4. In these cases, anaphylaxis is induced by minimally processed and unfermented soy, including soy milk, and symptoms maybe more profound when pollen counts are high.^4^

**Table I:** Popular grocery items containing soy

Brand Parent company, region sold	Product	Ingredient
Naked Juice PAI Partners, multinational	Protein smoothie	Soy protein
Dr Oetker Dr Oetker, multinational	Pizza crust	Soybean oil
Rustlers Kepak, multinational	Beef burger, processed meats	Hydrolyzed soy protein
Pizza Express Hony Capital, multinational	Beef pizza, chicken pizza	Soybean
Pot Noodle Unilever, multinational	Chicken-flavored noodles	Soybean
Batchelors Premier Foods, multinational	Chicken-flavored noodles	Soy sauce
Princes Mitsubishi, Europe	Chicken & Ham spread	Soy protein
Young’s Seafood Sofina foods, Europe	Seafood sticks	Soy protein
Quorn Monde Nissin, multinational	Vegetarian ready meal	Soybean
Mr Kipling Premier foods, multinational	Battenberg cake	Soy flour
Kingsmill Associated British Foods, multinational	Sliced white bread	Soy flour
Doritos PepsiCo, multinational	Chillwave tortilla chips	Hydrolyzed soy protein
Alpro Dean Foods, multinational	Yogurts, custard, desserts	Soybean
Cathedral City Saputo Dairy, Europe	Plant-based “cheese” spread	Soy protein
Danone MFS Investment, multinational	Desserts	Soy protein
Innocent Smoothie Coco Cola, multinational	Protein smoothie	Soy protein
Hovis Endless LLP, Europe	White bread	Soy flour
Kellogg's Kellogg, multinational	Granola	Soy flour
Bird's Eye Nomad Foods, multinational	Vegetable stir fry, Plant-based “chicken” and “beef”	Soybean, soy protein
Heinz Kraft Heinz, multinational	Spaghetti Bolognese	Soy protein
Warbutons Warbutons, Europe	White bread, pita bread, bagels	Soy flour
Walkers PepsiCo, multinational	Monster Munch corn snacks	Hydrolyzed soy protein
Nestlé Swiss, multinational	Plant-based chocolate drink	Soy protein
Cadbury Mondelez, multinational	Chocolate cake	Soy flour

Information extracted from publicly available NiQ consumer intelligence data.^5^

Soy is a protein-rich legume native to East Asia that is consumed under many guises, with fluctuating allergenicity owing to processing, heating, and fermentation (Table II).^6^ Soy has become more commonplace in Western diets because of multiculturalism and movement toward plant-based foods for health and environmental reasons.^7^ Although this trend is intentional, it is paralleled by the presence of soy as an inexpensive bulking product and thus, as a hidden allergen in many manufactured foods. Popular foods that contain soy range from processed meats and meat mimics to packaged bread and protein-enhanced snacks marketed around exercise^5^ (data sourced from the consumer intelligence company NiQ [Table I]).

**Table II:** Sources of soy foods categorized by processing method

Food product	Reported allergenicity, if known
Unfermented soy (Gly m 4 present, some denatured by heating; some Gly m 5 and Gly m 6 present)	
Edamame, niamame, soybeans; sprouts Immature soybean eaten raw or lightly cooked	Minimal processing, case reports of allergy
Soy flour; kinako Ground full-fat, dehulled, soy beans; roasted soy flour	Minimal processing, case reports of allergy
Soy milk Filtered liquid from ground cooked soybeans	Minimal processing, case reports of allergy
Soybean sprouts Sprouted soybean seeds	Minimal processing, case reports of allergy
Tofu, yakidofu, yuba Coagulated soy milk, lightly boiled tofu, dried tofu skin	Minimal processing, case reports of allergy
Fermented soy (most Gly m 4 denatured; Gly m 5, Gly m 6 present)	
Miso Fermented soybean paste with/without barley, rice	Small quantities soybean allergens; allergenicity depends on brand and fermentation
Natto Fermented soybeans	Whole soybean; case reports of allergy
Soy/shoyu sauce; tamari Fermented soybean, wheat, yeast, salt; gluten-free soy sauce	Most allergens denatured; few cases of allergy Avoided by patients with celiac disease and wheat allergy
Tempeh Fermented tofu	Similar to tofu, but allergenicity altered by fermentation; case reports of allergy
Secondary soy products (component allergen content not documented; common hidden triggers)	
Soy protein Ground defatted, dehulled soybeans	Protein-rich, case reports of allergy
Hydrolyzed soy protein Soy protein broken into smaller amino acid, present in processed foods	Protein-rich, case reports of allergy
Soya protein isolate Refined soy protein >90% protein, present in fitness supplements	Protein-rich, case reports of allergy
Soy oil Extracted from soybean seeds, widely used in cooking and present; isotretinoin used to treat acne vulgaris	Low protein content, tolerated by most patients with soy allergy
Soy lecithin Byproduct of soybean oil, extensively used emulsifier in manufactured foods	Negligible protein content, tolerated by most patients with soy allergy
Textured soy protein, textured vegetable protein Defatted soy flour product	Protein-rich, variable protein profile and allergenicity

Alternative names, description of food, and reported of allergenicity, if known.^6^

It is evident from the literature that soy allergy and SDEIA reactions are often misinterpreted as a case of inadvertent contamination of a food product with peanut, with the correct diagnosis revealed after multiple, further episodes or regrettably at the postmortem examination.^8^ In Sweden, there have been 4 reported deaths in children with peanut allergy on account of non–exercise-associated soy anaphylaxis; all of the children had been unaware of their soy allergy, and all of the fatal events were triggered by processed meat products^8^ (eg, meatballs, hamburger, kebab). It is thought that up to 35% of patients with peanut allergy are soy sensitized.^9^

Soy protein is seemingly ubiquitous, present in more than one-third (36%) of the United Kingdom’s most popular supermarket brands, most of which are sold globally^5^ (Table I). Legislation in the United States and Europe ensures that soy (interchangeable with soya) is declared as a major allergen on all manufactured foods; however, because of its hidden presence, variable nomenclature, and changeable allergenicity, neither the patient nor the clinician may realize that the allergen was present and consumed. Furthermore, labeling soy lecithin–containing products as containing soy further confuses matters and potentially places needless restrictions on patients. Soy lecithin is an emulsifier derived from highly processed soy oil with minimal soy protein (100-500 ppm) and is present in almost two-thirds (64%) of popular grocery items.^5^ Although most patients with IgE-mediated soy allergy can and do tolerate soy lecithin, the US Food and Drug Administration continues to list it as an allergen owing to the low amount but presence of soy protein.^10^ Therefore, despite international bodies identifying soy lecithin as safe in patients with soy allergy, this fact is not being reflected on product labels.

Our case demonstrates that soy allergy and SDEIA can present with reactions to what appear to be disparate foods. The exercise element also calls attention to another emerging risk, as soy-enhanced foods are increasingly being marketed to exercise enthusiasts, thus providing the perfect storm for SDEIA events. Early recognition of anaphylaxis and prompt use of adrenaline can be lifesaving in these patients; however, identification of the culprit food is crucial to avoiding further episodes.

## Disclosure statement

Disclosure of potential conflict of interest: The authors declare that they have no relevant conflicts of interest.
